# Assessment of the Piezoelectric Response of an Epoxy Resin/SbSINanowires Composite Filling FDM Printed Grid

**DOI:** 10.3390/ma13225281

**Published:** 2020-11-22

**Authors:** Mateusz Kozioł, Piotr Szperlich, Bartłomiej Toroń, Piotr Olesik, Marcin Jesionek

**Affiliations:** 1 Faculty of Materials Engineering, Department of Advanced Materials and Technologies, Silesian University of Technology, ul. Krasinskiego 8, 40-019 Katowice, Poland; piotr.olesik@polsl.pl; 2 Institute of Physic—Centre for Science and Education, Silesian University of Technology, ul. Krasinskiego 8, 40-019 Katowice, Poland; piotr.szperlich@polsl.pl (P.S.); bartlomiej.toron@polsl.pl (B.T.); Marcin.Jesionek@polsl.pl (M.J.)

**Keywords:** epoxy resin, nanocomposite, nanosensors, antimonysulphoiodide, fuse deposition modeling

## Abstract

This paper shows a piezoelectric response from an innovative sensor obtained by casting epoxy-SbSI (antimony sulfoiodide) nanowires nanocomposite to a grid structure printed using a fuse deposition modeling (FDM) method. The grid is shown to be a support structure for the nanocomposite. The applied design approach prospectively enables the formation of sensors with a wide spectrum of shapes and a wide applicability. The voltage signal obtained as a result of the piezoelectric effect reached 1.5V and 0.5V under a maximum static stress of 8.5 MPa and under a maximum dynamic stress of 22.3 kPa, respectively. These values are sufficient for potential application in sensor systems. The effect of a systematic increase in the voltage signal with subsequent cycles was also observed, which similarly allows the use of these sensors in monitoring systems for structures exposed to unfavorable cyclical loads. The obtained results also show that the piezoelectric signal improves with increase in strain rate.

## 1. Introduction

Strain sensors are used to monitor the behavior of a variety of advanced structures, including load-bearing elements in aviation [1]. They form systems which together are used for structural health monitoring (SHM). The most commonly used solutions are vibrating wire [2], optical [3], piezoresistive [4] or piezoelectric sensors [5] mounted to the construction or embedded into the material’s structure. Systems based on vibration and noise analysis are used [6,7]. The main purpose of using monitoring systems is to ensure the safety of the structure by registering dangerous overloads as well as identifying possible causes of the hazardous state [8,9]. Monitoring systems are also used, for example, for the analysis of molding processes [10,11]. Usually, inserting sensors into the material structure disrupts its local continuity, weakening the structure at the place of insertion [12]. Sometimes, however, this impact may be insignificant, as exemplified by our previous work [13], where the SbSI-based sensor introduced into the glass fiber reinforcing polymer (GFRP) composite structure caused only a slight weakening, comparable to standard functional modifications of composite structures [14,15]. Conversely, placing the sensors outside the structure of the material may translate into a lower accuracy of measurements, as the deformation state could differ significantly through different parts of the loaded cross-section [16,17].

Fuse deposition modeling (FDM) is currently the most popular method of 3D printing. It is characterized by a high versatility, relative simplicity, and a low price for both the tooling and batch materials. The method is suitable, to a limited extent, for the direct printing of some types of sensors, such as resistance force/strain sensors based on conductive thermoplastic composites containing carbon nanotubes [18,19]. A more frequent problem concerning FDM printing and sensors is the placement of sensors inside the printed structures, i.e., covering various types of sensors with printed surroundings [20,21]. No successful attempts have been made so far to print a piezoelectric sensor directly, which is due to difficulties in obtaining a material both printable and piezoelectric at the same time. This study introduces an alternative approach to the two methods described above. It was decided to use a printed grid as a support structure for casting piezoelectrically active nanocomposite materials that—after being cast and cured—could function as a strain sensor. In such a case, the shape of the sensor depends on the shape of the grid and the nanocomposite filling of the grid gives the sensor functionality.

Our previous work [13] showed a piezoelectric sensor based on a composite of epoxy resin and SbSI nanowires, placed as a thin layer inside the structure of glass fiber reinforcing a polymer (GFRP) laminate structure. A satisfactory electrical response signal generated during the bending of the sample over the elastic strain range of the composite was obtained. The sensor was a thin (0.05 mm) layer. In the conclusions of the article [13], we questioned whether increasing the thickness of the sensor would significantly change the obtained signal, or whether the thickness would adversely affect the signal due to the high specific resistance of the nanocomposite used.

The purpose of this work is to produce and evaluate an innovative deformation sensor. The sensor is constructed from a 3D (FDM) printed skeleton grid filled with nanocomposite of epoxy resin filled with SbSI nanowires. The evaluation included preliminary tests of voltage response during static bending. The printed skeleton provides the shape and dimensions of the sensor, which theoretically has a very wide range. Results of the tests are to show whether such constructed sensor works and how does it work.

The novelty included in this study is the determination of whether a sensor similar to that developed in study [13], but with greater thickness, will work and whether the value of the voltage signal will increase (due to the increase in thickness).The big advantage of the studied sensor based on the printed grid is expected possibility of its shaping to various shape and dimensions. It is relatively elastic, in opposite to competitive materials, e.g., quartz.

## 2. Materials and Methods

### 2.1. Manufacturing of the Sensor

The sensor was produced according to the following scheme:

(1) Printing a grid using the FDM method.
(2) Preparing a composite mixture of resin containing SbSI nanowires.
(3) Applying the mixture to the grid and curing it.

**Figure 1:**
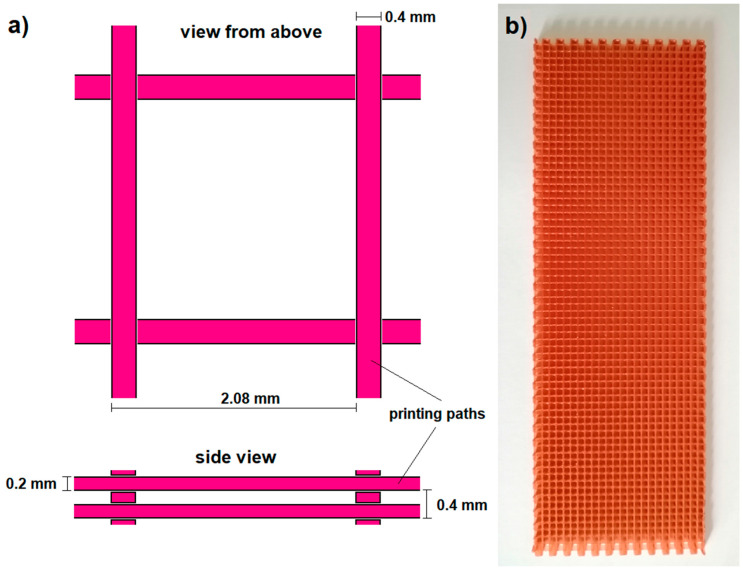
The printed grid: (**a**) a scheme of the arrangement of paths in a single “cell” of the printout, (**b**) an exemplary printed grid with dimensions of 150 × 50 mm.

The grid printing was completed using a PRUSA MK3S printer (Prusa, Prague, Czech Republic) with a path height of 0.2 mm and a path thickness of 0.4 mm. The printing material used was polylactide (PLA) from COLORFIL, Sosnowiec, Poland. The infill ratio of the printout was 25 %, a grid was printed without external walls, the printed grid and the print scheme are given in Figure 1.

**Figure 2:**
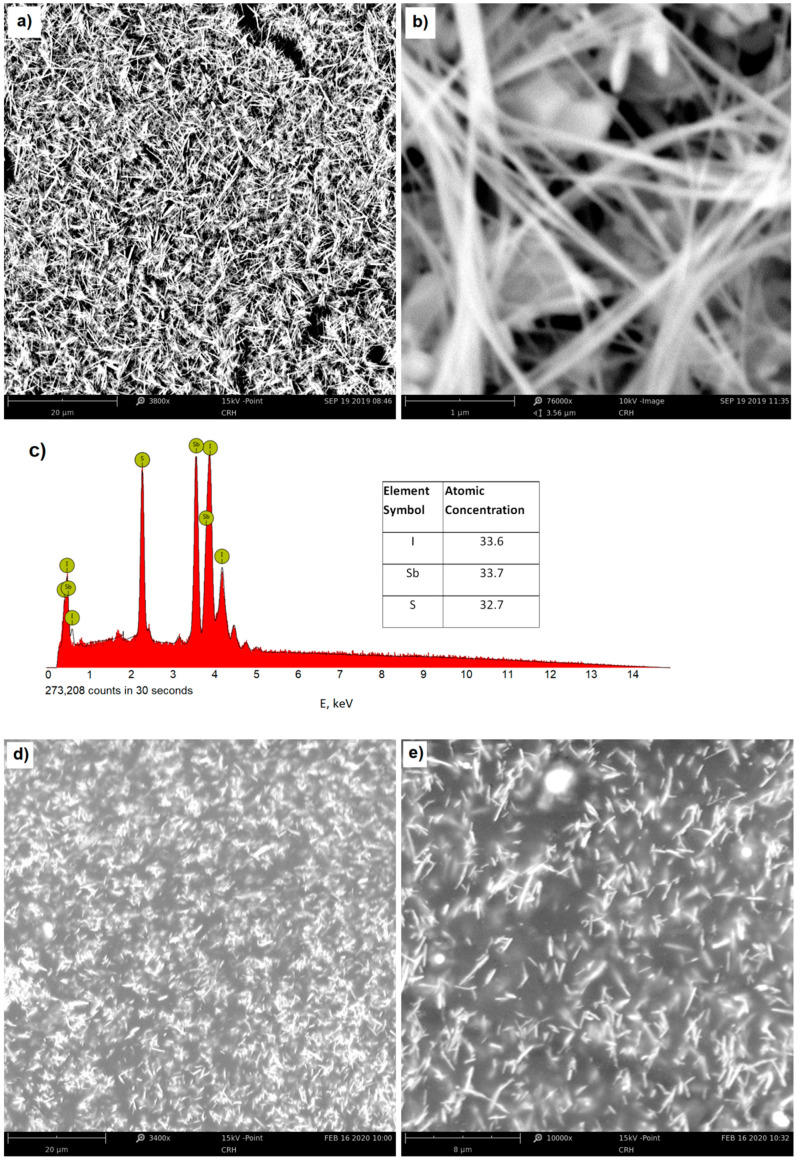
The SEM micrographs (**a**,**b**) and EDS spectrum (**c**) of SbSI nanowires—the inset table shows the atomic concentrations of elements, (**d**,**e**) SEM micrographs of the obtained nanocomposite of epoxy resin with SbSI nanowires.

The SbSI nanowires were prepared by sonochemical synthesis using a sonotrode from stoichiometrically dosed pure components (antimony, sulfur, and iodine). The ultrasound frequency during the process was 20 kHz and the power density was 565 W/cm^2^. The obtained raw material was then washed in ethanol, which gave nanowires in the form of a powder xerogel. The nanowires had lateral dimensions of 10 nm to 50 nm and a length up to several micrometers. Complete details concerning the character and production process of the SbSI xerogel have been previously presented in [22,23]. Figure 2a–c show the enlarged micrographs of the nanowires and the composition analysis obtained by energy dispersive spectroscopy (EDS), using a Phenom PRO X scanning electron microscope (SEM) by Thermo Fisher Scientific (Waltham, MA, USA) equipped with an energy-dispersive X-ray spectroscopy (EDS) detector. Figure 2d,e present the SEM images of the obtained nanocomposite. They show a homogeneous distribution of nanowires throughout the entire volume of the resin constituting the matrix of the nanocomposite, and a practical lack of bundles and local densities.

A detailed description of the production of the resin, SbSI nanowires powder composite has already been described in detail [13]. Table 1 presents a comparison of piezoelectric properties of SbSI and other materials.

The manufactured nanowires were added to the LH288 epoxy resin (HAVEL COMPOSITES, Svésedlice, Czech Republic) in an amount of 20 g per 64 g of the resin. The mixture was mixed mechanically and then treated using an ultrasonic scrubber Elmasonic S 750 Watt (ELMASONIC, Łomża, Poland) for 60 min. After preparing the mixture, 16 g of the hardener H281 (HAVEL COMPOSITES, Svésedlice, Czech Republic) was added (proportion of resin to hardener 4:1) and then mixed mechanically. After addition of hardener, the mixture remains liquid for approximately 25 min (working lifetime). The amount of SbSI in the resin with hardener was 20 wt%. During preliminary research, it was found that the addition of SbSI in an amount of 20 wt% was optimal. An amount lower than 20 wt% does not guarantee a stable piezoelectric effect, an amount greater (even 40 wt% has been tried) does not improve the effect and even worsens its stability and repeatability.

The prepared material was poured into the printed grid placed in a plastic mold (size 150 × 50 × 3 mm, made of PLA). The grid was filled with the nanocomposite by gravity casting. The resin curing process is associated with the heat generation and temperature increase. This should be taken into account when the volume of the cross-linked material is greater. In the analyzed case, the temperature control on the surface of the curing mass did not exceed 70 °C. Even excessive local temperature fluctuations that could affect the mechanical properties of the material used at any point in the volume are unlikely in the analyzed case [28]. No deformation of the grid was observed, and special attention was given to preventing this, as the softening point of the PLA used for the grid was 60 °C. Moreover, the thermal decomposition of SbSI only occurs at a temperature of 270 °C (see Figure A1 in the appendix of [29]); thus, for the described case, the curing temperature did not affect the crystal structure and thus the piezoelectric properties of the SbSI nanowires.

**Figure 3:**
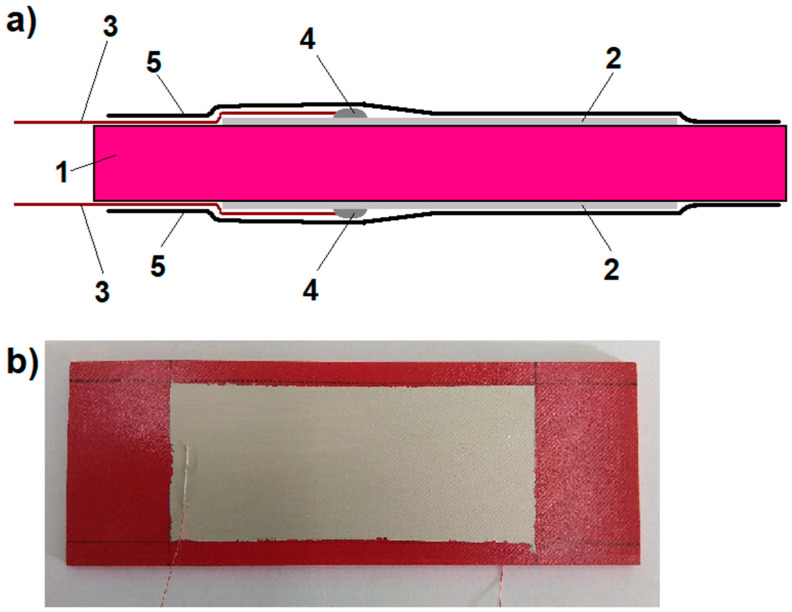
The prepared sample of the piezoelectric composite applied to the PLA grid core: (**a**) a front-view cross-section schema showing the arrangement of the individual elements on the sample surface; 1—the piezoelectric composite on the grid core, 2—silver electrode, 3—copper wire outputting a signal, 4—electrode-wire connection point, and 5—external PVC securing tape, (**b**) a photo of atop of the sample before securing it with PVC tape.

After removing the cured plate from the mold, it was found that the grid was completely covered with the piezoelectric composite, creating a 0.5 mm layer on the outer print paths. Then, silver electrodes with dimensions of 40 × 100 mm were applied to the plate on both sides using silver paste (05002-AB from SPI Supplies, West Chester, PA, USA). Using the same paste, copper wires were attached to the electrodes, which led the signal to the measuring system. The whole sample was finally covered with a protective insulating polyvinyl chloride (PVC) tape. A schematic diagram of the prepared sample is shown in Figure 3.

### 2.2. Sensor Testing

The produced samples of plate-sensors were tested basically using two methods: (1) static non-destructive bending over the elastic strain range of the material, the method analogous to that used in [13] for testing sensors integrated with laminates, and (2) a dynamic method with the use of an electromagnetic shaker.

**Figure 4:**
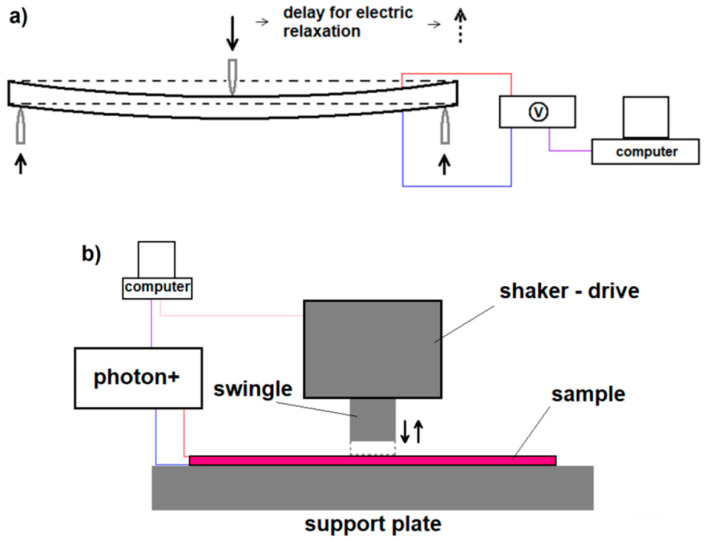
The scheme of: (**a**) measuring the voltage signal during static non-destructive bending of the sample; (**b**) experimental set-up of the dynamic tests with the use of a shaker.

The static bending was performed using an INSTRON 4469 testing machine (Instron, Norwood, MA, USA). The test consisted of bending the sample in a 3-point system to a specified deflection value (max. 3 mm), not exceeding the elastic strain range for the sample material with a defined deflection speed (from 1 to 500 mm/min). Maximum stress generated in a sample was 8.5MPa.The spacing of the supports was 140 mm. During bending, the voltage signal generated by the samples was measured. During the voltage measurement, a 120 s wait until a constant value of the measured voltage was established (sample loading), and the system returned to its initial state (sample unloading). The 120 swas based on preliminary tests for small deflections and low strain rates. This guaranteed the value of the recorded signal would be fixed. A Keithley 6517B voltmeter (KEITHLEY, Solon, OH, USA) was used for the electrical measurements. A schematic image of the bending test is shown in Figure 4a.

The dynamic tests were performed using an LDS V201 shaker (Bruel&Kjaer, Nærum, Denmark). During the tests, the sample was rigidly mounted on the support plate and loaded by hitting the central part of the swingle (free from the connection point of the signal output wires). The striking area of the circular swingle is 5 cm^2^. During the tests, the voltage signal and the corresponding acceleration of the swingle strokes in the sample were measured and are schematically illustrated in Figure 4b. A Photon + oscilloscope card (Bruel&Kjaer, Nærum, Denmark) and the Delta Tron Type 4507 B 001Accelerometer (Bruel&Kjaer, Nærum, Denmark) were used. The impact cycle frequency was tested in the range of 3–2000 Hz. The maximum stress generated in a sample was 22.3 kPa.

## 3. Results and Discussion

**Figure 5:**
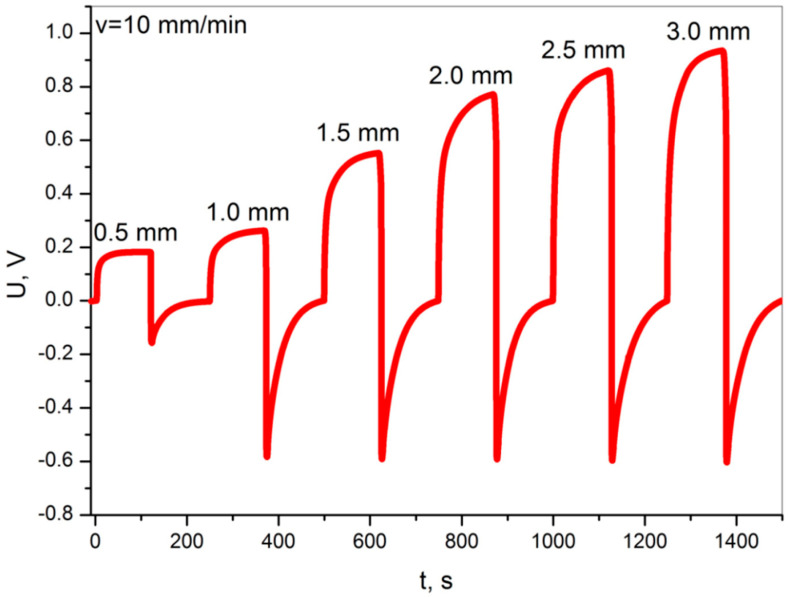
Voltage diagrams obtained during the non-destructive static bending tests at a constant bending rate 10 mm/min, and with a variable deflection of 0.5–3.0 mm.

Figure 5 shows the voltage graphs recorded during the non-destructive static bending of the specimen, at a constant bending rate of v = 10 mm/min, successively for increasing deflection values in the range of 0.5–3.0 mm.

**Figure 6:**
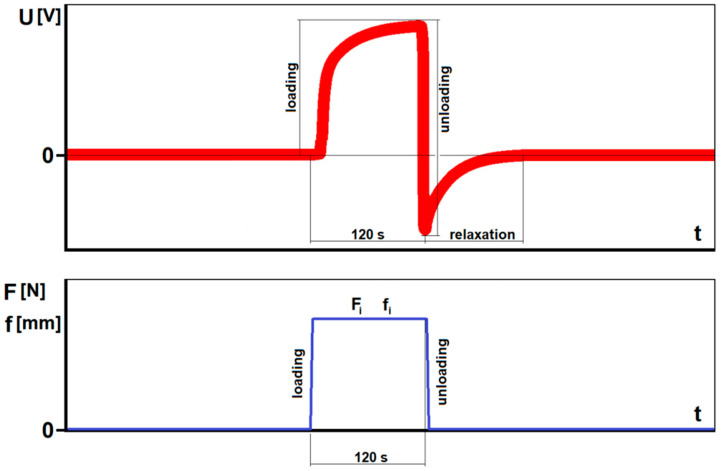
Explanation of the single voltage course and corresponding loading-unloading cycle for non-destructive static bending of the specimen: F—load, f—deflection, f_i_—assumed value of deflection, F_i_—a load value corresponding with the f_i_ value (within the elastic conditions a load is directly proportional to a deflection).

The presented graph (Figure 5) consists of cyclically repeating signals corresponding to a single non-destructive bending of the sample. At the beginning of each measuring cycle, the sample is unloaded. The measurement begins with loading to a specified deflection, at a specified speed of the loading pin (bending rate). After loading, the sample is held under load (deflected) for 120 s. Then the sample is unloaded to its starting position and is allowed to discharge electrically (relaxation) until the voltage reaches 0. The loading-unloading cycle is illustrated in Figure 6.

**Figure 7:**
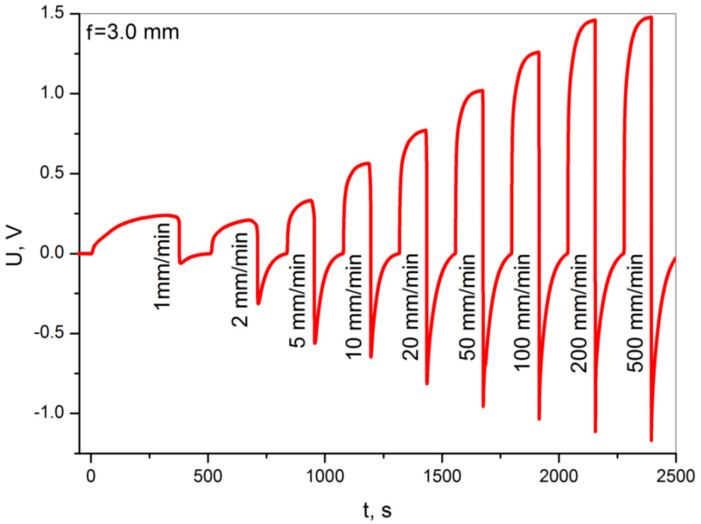
Voltage diagrams obtained during the non-destructive static bending tests at a constant deflection of 3.0 mm, with a variable bending rate of 1–500 mm/min.

Figure 7 shows voltage graphs for non-destructive static bending tests at a constant deflection of 3.0 mm and an increasingly higher bending rate over the range of 1–500 mm/min.

The obtained voltage results show 1.0 V for higher deformation at low speed (Figure 5) and about 1.5 V for high speeds (Figure 7), which are considered favorable for structural deformation applications. The voltage increases are distinct and repeatable. The obtained voltage level enables the potential use of these sensors in various applications related to the monitoring of structural deformation. The fact that the sample discharges with a large time constant, with appropriate signal processing, will allow one to determine the amplitude but and rate of deformation.

**Figure 8:**
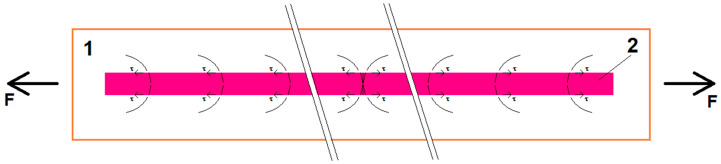
Nanowires as a continuous reinforcing element receiving the load from the matrix through tangential stresses; τ: 1—matrix (cured resin), and 2—nanowire connected to the matrix.

Increasing the obtained voltage increases the maximum deflection of the sample, which is in line with expectations. This results from an increased deformation of the nanowires filling the polymer matrix, which translates into a progressive piezoelectric effect [30]. It is more difficult to explain the issue of the voltage increase with the increase in the strain rate, with constant maximum deflection. The obtained effect is repeatable and irrefutable. However, there are no described phenomena in the literature that would allow this observation to be fully explained. Increasing the deformation rate of the sample is connected with increasing the internal mechanical resistance of the material that forms it, and by increasing the value of the elastic modulus [31,32] with increasing the stress. The nanowires, as an element filling the composite, should be treated as a continuous fibrous reinforcement. Assuming its good connection with the resin-matrix, it will receive the load by shear stresses, following the theory of composites [33,34], see Figure 8.

**Figure 9:**
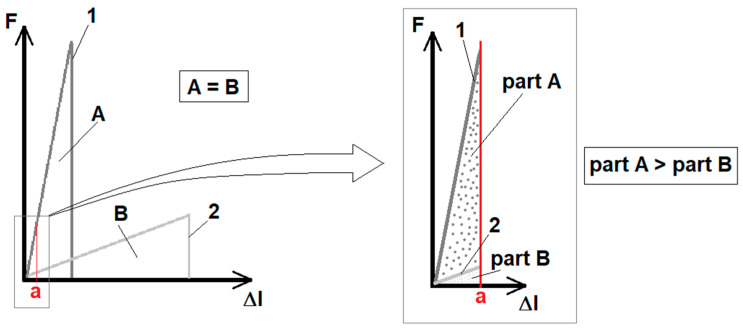
Schematic illustration of the influence of the strain rate on the resistance energy of a loaded material: 1—F-Δl curve for a brittle and rigid (without plastic deformation) material loaded with a high strain rate, 2—F-Δl curve for a similar sample of the same material loaded with a lower strain rate; A—the energy of destruction of a sample deformed at a higher speed, B—energy of destruction of a sample deformed at a lower speed; part A—deformation energy of the sample deformed at a higher speed on the displacement section limited by a value of a, part B—strain energy of a sample deformed at a lower speed over the displacement distance limited by a value of a.

Increasing the strain rate is therefore definitely associated with increasing the stress in the nanowires during deformation, but without increasing the maximum achieved strain. At the same time, at a higher strain rate, a greater amount of energy will be introduced into the sample per unit time than at a lower speed as outlined in Figure 9.

Assuming a good connection between the matrix and the nanowires, we propose that the nanowires act as reinforcing elements of the composite structure and will create greater mechanical resistance against the external action, which by a specific mechanism transfers into the intensity of the generation of electric charges and the value of the generated voltage. Of course, the energy introduced to the sample by the testing machine at the material’s elastic strain range is theoretically fully returned when the material is unloaded. In practice, however, even during elastic (non-remaining) deformation, some of the energy dissipates, mainly as heat generated by internal friction of the material, but also to other effects such as electric energy generated by the piezoelectric effect. The observed influence of strain rate on the obtained voltage should be analyzed in more detail at the molecular level, as there is a dearth of information about this effect in the literature. This is beyond the scope of this study and will form part of a further molecular level study.

The trends of the obtained results are analogous to those obtained in the work [13] for sensors integrated in the laminate structure. On the other hand, when referring the obtained voltage values to the results of work [13], it should be stated that they are much higher. However, taking into account the size of the electrode surface (in this paper it is 40 cm^2^, and in paper [13] it was 6 cm^2^) and assuming that the signal value is proportional to it, the results obtained in the two studies are comparable for the selected similar conditions. This confirms the thesis that the thickness of the piezoelectrically active area does not affect the size of the generated signal in the tested type of sensors. This means that the electrical signal received by the electrode is generated in the subsurface areas adjacent to the electrode and no charge is displaced from deeper areas of the sensor. This is probably due to the relatively high specific resistance of cured resin used as a matrix in the nanocomposites. It also indicates that there are no significant nanoeffects related to conduction (inductive or tunnel) that could theoretically be expected in the nanocomposite, especially at a high concentration of nanocomponents [30,31].

**Figure 10:**
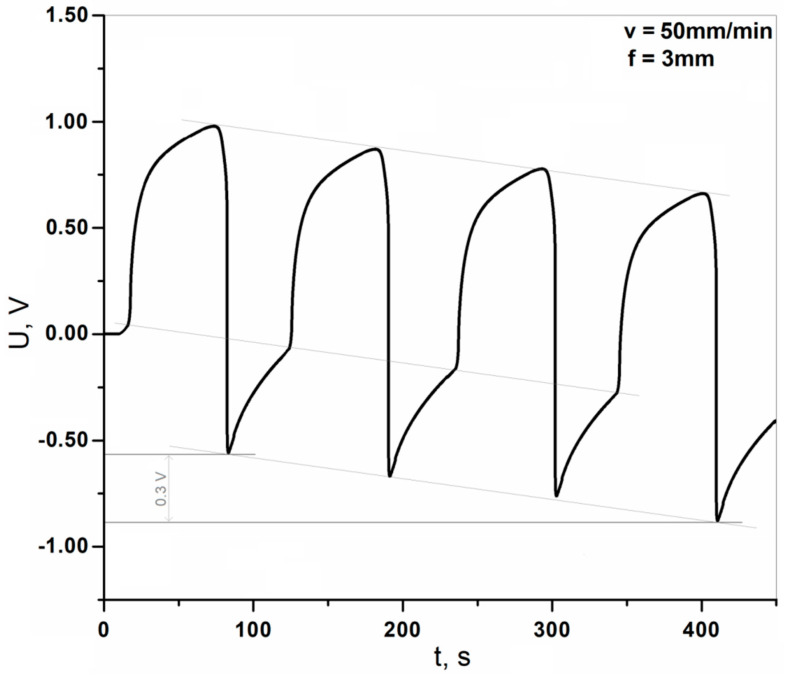
Voltage diagrams obtained during non-destructive static bending tests without waiting for sample discharge (deflection of 3.0 mm, bending rate of 50 mm/min).

There is one more effect observed during the tests that should be mentioned. When, after loading the sample, waiting, and then unloading, and another loading cycle was performed only after 50 s—before the sample was fully discharged—it was noticed that with each successive cycle the voltage value increased (voltage in fact decreased, but the absolute value increased)—Figure 10.

In Figure 10, we can observe that for consecutive cycles, this change of peak voltage is practically linear for several characteristic points of the plot. The recorded voltage “shifts” down with each successive cycle. It should be emphasized that this effect is reproducible. One assumes that this proposed sensor could serve as a structure monitoring system which warns the operator about unfavorable cyclic deformations repeated over undesirably short periods. The registration of exceeding a certain voltage value is much less complicated than the registration and analysis of periodically repeating signals.

The waveform of the voltage signal shows no significant influence on the viscoelasticity of the materials. The grid supporting structure spread across the entire sample volume is a thermoplastic polymer. Additionally, the nanocomposite consists of a cured polymer resin that exhibits a certain range of viscoelastic deformations that disappear over time [35,36]. Signal disturbances could be expected, mainly due to stress relaxation [32,33]. However, it is not possible to distinguish the voltage effects on the diagrams in Figure 5 and Figure 7 that would indicate the effect of viscoelasticity.

Comparison of the sensor with other competitive ones is not simple task, because of specific load conditions—the signal was obtained in bending tests under the conditions of variable stress in the cross-section, according to the theory of beam bending [37]. The value of tensile and compressive stress at the maximum applied deflection was continuously variable from 0 to approximately 8.5 MPa (average 4.25 MPa). This produced close to1.5V voltage. For comparison, a quartz piezoelectric sensor at a pressure of approximately 5.5 MPa gives a signal of 1.8 V [38]. Precise load sensor with a sandwich structure,-based mainly on PZT (lead zirconatetitanate) and quartz, planned among others for medical applications, was described in [39]. It shows 11.5 mV at a pressure of 30 kPa. Converting proportionally, this structure would give 1.7V at a pressure comparable to ours. It should also be emphasized that the surface area of the electrodes used in the study referred to was smaller than in our case. This means that the structure used in [39] results in an approximately comparable output signal as the nanocomposite tested in this study. Of course, a more reliable comparison of the tested nanocomposite with competing piezoelectrics will be the subject of further research. The SbSI nanowires have been compared with other piezoelectric materials in several former studies (e.g., [29,40]).

**Figure 11:**
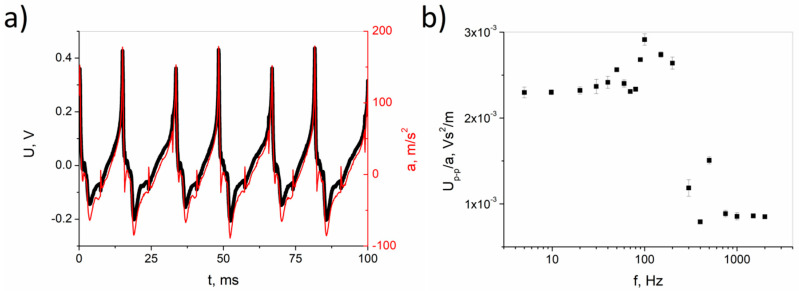
Dynamic loading tests using a shaker with acceleration and generated voltage at a frequency of 60 Hz (**a**), and the frequency response sensitivity of the nanosensor (**b**).

The manufactured sample was also tested under cyclical dynamic loads, with the use of a shaker. A graph showing a series of measurements conducted at a frequency of 60 Hz (optimally determined in the initial tests) is shown in Figure 11a. Figure 11b shows the peak-to-peak voltage normalized by the acceleration of the piezoelectric nanosensor for miscellaneous excitation frequencies.

Under dynamic conditions, the sample shows a significant voltage signal. However, no signal overlap is observed (as in Figure 10). This is likely due to the part of the sample subjected to deformation is smaller here than in the non-destructive bending tests. This results in a much faster piezoelectric response and a shorter time needed to discharge the generated charge. It can be seen that the highest sensitivity of the sample occurs for frequencies lower than 200 Hz, reaching 2.5 × 10^−3^ Vs^2^/m. This is essential for the construction of usable devices for failure structure monitoring.

It should be emphasized that the tested sample did not suffer any damage or any permanent deformations, despite the numerous static and dynamic tests. The created structure combining a grid printed from thermoplastic material and the nanocomposite based on epoxy resin is a highly promising solution for sensors with a complex shape. The behavior at low/high temperatures, resistance to abrasive wear, and the impact resistance of the nanocomposite sensors on printed grids will be the subject of further research.

The concept of current conduction in non-metallic nanocomposites remains a problem. In the case of a mixture of a nano-semiconductor with a non-conductive substance (such as a polymer resin), the current generation effect is probably limited to areas near the connecting surface of such a nanocomposite which have the signal receiving electrode. This means that the tangential stresses resulting from shear [34] are of greater importance for the signal generation at the sensor placed in the bending beam, compared to normal stresses. The influence of the loading arrangement of the sensor on the generated electric signal will be the subject of further research. The results of this research will expand our knowledge about the applicability of the epoxy-SbSI-based sensors.

## 4. Conclusions

A sample containing a nanocomposite of epoxy resin with SbSI nanowires, cast to a thermoplastic grid printed using the FDM method, was tested by piezoelectric excitation tests. The obtained results allowed us to draw the following conclusions:

- Thermoplastic grid printed using the FDM method works well as a load-bearing structure for an epoxy resin composite containing SbSI nanowires. This provides the desired shape with appropriate stiffness and elasticity, and at the same time susceptibility to introduced deformation. This method may turn out to be a way of forming sensors with a wide spectrum of shapes, enabling a wide application; however, this aspect requires further research.
- The voltage signal obtained in the tested sample, as a result of the piezoelectric effect, reaches a level of 1.5 V over a range of elastic (non-remaining) deformations of the material and is sufficient for potential application in sensor systems.
- The sample showed a very clear piezoelectric effect. The voltage above 0.5 V was obtained under dynamic loading conditions at maximum stress of 22.3 kPa. This is particularly suitable for monitoring structures subjected to deformations with a frequency up to 200 Hz.
- In the case of repeated static loads with a frequency that prevents the sample from being discharged after each cycle, the effect of a systematic increase in the voltage signal with subsequent cycles was observed. This effect is reproducible and predictable. It enables the potential use of sensors similar to the one tested in systems monitoring structures exposed to unfavorable cyclical loads.
- Comparing the obtained results to previous research work, it should be stated that the thickness of the sensor has a small, disproportionate effect on the strength of the signal obtained, which supports the thesis that the piezoelectric signal in sensors of this type is generated in areas near the surface at the nanocomposite contacts with the electrodes.

## Figures and Tables

**Table 1:** Comparison of piezoelectric properties of SbSI and other materials: d_33_—piezoelectric coefficient, k_33_—electromechanical coupling factor (into “33” direction).

Material	d_33_ [pC/N]	k_33_
SbSIsingle crystal	1000 [24]	0.9 [24]
PZT-5	375 [25]	0.61 [25]
95% BaTiO_3_ 5% CaTiO_3_	149 [26]	0.48 [26]
BaTiO_3_	73 [27]	0.52 [27]
LiNbO_3_	6 [25]	0.17 [25]
Quartz	2.0 (d_11_) [26]	0.09 [26]
